# Polypurine reverse hoogsteen hairpins as a therapeutic tool for SARS-CoV-2 infection

**DOI:** 10.1016/j.jbc.2024.107884

**Published:** 2024-10-11

**Authors:** Carlos J. Ciudad, Simonas Valiuska, José Manuel Rojas, Pablo Nogales-Altozano, Anna Aviñó, Ramón Eritja, Miguel Chillón, Noemí Sevilla, Verónique Noé

**Affiliations:** 1 Department of Biochemistry & Physiology, School Pharmacy and Food Sciences, Universitat de Barcelona, Barcelona, Spain; 2 Institut de Nanociencia i Nanotecnologia (IN2UB), Universitat de Barcelona, Barcelona, Spain; 3 Centro de Investigación en Sanidad Animal-CISA, INIA, CSIC, Madrid, Spain; 4 Institute for Advanced Chemistry of Catalonia, CSIC, Barcelona, Spain; 5 Centro de Investigación Biomédica en Red de Bioingeniería, Biomateriales y Nanomedicina, Instituto de Salud Carlos III, Madrid, Spain; 6 Institute of Neurosciences, Universitat Autònoma de Barcelona, Barcelona, Spain

**Keywords:** PPRH, SARS-CoV-2, therapy, oligonucleotide, pandemic

## Abstract

Although the COVID-19 pandemic was declared no longer a global emergency by the World Health Organization in May 2023, SARS-CoV-2 is still infecting people across the world. Many therapeutic oligonucleotides such as ASOs, siRNAs, or CRISPR-based systems emerged as promising antiviral strategies for the treatment of SARS-CoV-2. In this work, we explored the inhibitory potential on SARS-CoV-2 replication of Polypurine Reverse Hoogsteen Hairpins (PPRHs), CC1-PPRH, and CC3-PPRH, targeting specific polypyrimidine sequences within the replicase and Spike regions, respectively, and previously validated for COVID-19 diagnosis. Both PPRHs are bound to their target sequences in the viral genome with high affinity in the order of nM. *In vitro*, both PPRHs reduced viral replication by more than 92% when transfected into VERO-E6 cells 24 h prior to infection with SARS-CoV-2. *In vivo* intranasal administration of CC1-PPRH in *K18-hACE2* mice expressing the human ACE receptor protected all the animals from SARS-CoV-2 infection. The properties of PPRHs position them as promising candidates for the development of novel therapeutics against SARS-CoV-2 and other viral infections.

Severe Acute Respiratory Syndrome Coronavirus 2 (SARS-CoV-2) belongs to the family of coronaviruses, which are enveloped, positive, and single-stranded viruses. This family includes viruses responsible for common colds as well as severe pathogens such as SARS-CoV and MERS-CoV (1, 2). SARS-CoV-2 is responsible for the COVID-19 outbreak that originated in Wuhan, China, in late 2019, and was declared as a global pandemic in March 2020 for its rapid spread and high fatality rate (3). In May 2023, the World Health Organization declared that COVID-19 was no longer classified as a public health emergency of international concern. SARS-CoV-2 infected over 704 million individuals and caused more than seven million deaths worldwide by April 2024 https://www.worldometers.info/coronavirus/coronavirus-cases/ (Accessed May 15, 2024). However, these numbers might be underestimated due to many non-detected asymptomatic cases.

The genetic material of SARS-CoV-2, whose RNA is about 30kb, carries instructions for the synthesis of both structural and non-structural proteins. The non-structural proteins include two open reading frames (ORF), including ORF 1a and ORF 1b, that are translated into two polyproteins, pp1a and pp1ab (4). Structural proteins consist of spike (S), membrane (M), envelope (E), and nucleocapsid (N) along with accessory proteins. Spike interacts with the human ACE2 receptor, allowing viral attachment and fusion with the membrane. Then, the viral genome is transcribed and translated by the host machinery, and the newly synthesized viral RNA and proteins are assembled in the host cells' cytoplasm. Finally, viral particles are enclosed in vesicles, transported to the cell surface, and released. This process frequently results in the programmed cell death of the infected cells (1, 5).

During the pandemic, the scientific community worked intensively to develop a wide array of therapies against SARS-CoV-2. The primary approach was to obtain an effective and large-scale producible vaccine. While the development of traditional vaccines typically spans a period of 10 to 15 years, COVID-19 vaccines were generated and authorized for their emergency use within a remarkably short timeframe, ranging from 12 to 16 months (6). In parallel, other options were considered to treat and reduce COVID-19 symptoms (7, 8). One approach was the usage of antiviral drugs such as polymerase or protease inhibitors, immune modulators, viral entry inhibitors, and neuraminidase inhibitors, which target various stages of the viral life cycle by interfering with specific viral proteins or enzymes (9, 10). Another approach was based on monoclonal antibodies specific against the SARS-CoV-2 spike protein (11). Other therapies include convalescent plasma therapy used, as a temporary protection, recovered plasma from previously infected patients, which provides antibodies against the virus (12) or corticoids as anti-inflammatory drugs to modulate the immune system in critical patients (13).

Alternative strategies to potentially repress viral replication involved the usage of therapeutic oligonucleotides to target SARS-CoV-2 sequences. Some of the strategies include antisense oligonucleotides (ASOs) (14, 15), microRNAs (miRNA) (16), small-interfering RNAs (siRNA) (18,19,20,21), or CRISPR-based systems (17, 18). In this work, we used Polypurine Reverse Hoogsteen (PPRH) hairpins targeting specific SARS-CoV-2 regions for therapeutic and protective purposes against the viral infection and its spread. PPRHs are non-modified single-stranded DNA molecules made of two polypurine strands, linked by a four-thymidine loop, that run in antiparallel orientation and interact with each other by Hoogsteen bonds, adopting a hairpin conformation. These molecules are designed to specifically bind by Watson-Crick bonds to a DNA or RNA sequence rich in polypyrimidines and to form a triplex structure, displacing the complementary strand in the case of dsDNA (19, 20, 21, 22). The target sequence does not have to be a pure polypyrimidine stretch and can present up to three purine interruptions. Thus, PPRHs can be designed to target practically any gene in the genome (23). Furthermore, PPRHs show great stability in serum and cultured cells (24). Our research group has previously designed PPRHs directed towards SARS-CoV-2 for diagnostic purposes, namely CC1-PPRH, CC2-PPRH, and CC3-PPRH, targeting the *replicase*, *N gene*, and *spike* regions of the SARS-CoV-2 genome, respectively (25). From the stability analyses of the triplexes formed by these three PPRHs, it was found that CC2 had a lower binding affinity to its target (26). For this reason, CC2 was excluded from the present work and we focused on CC1 and CC3 PPRHs to evaluate their potential therapeutic effect against the virus both *in vitro* and *in vivo*.

## Results

### PPRH target selection and sequence design

**Figure 1:**
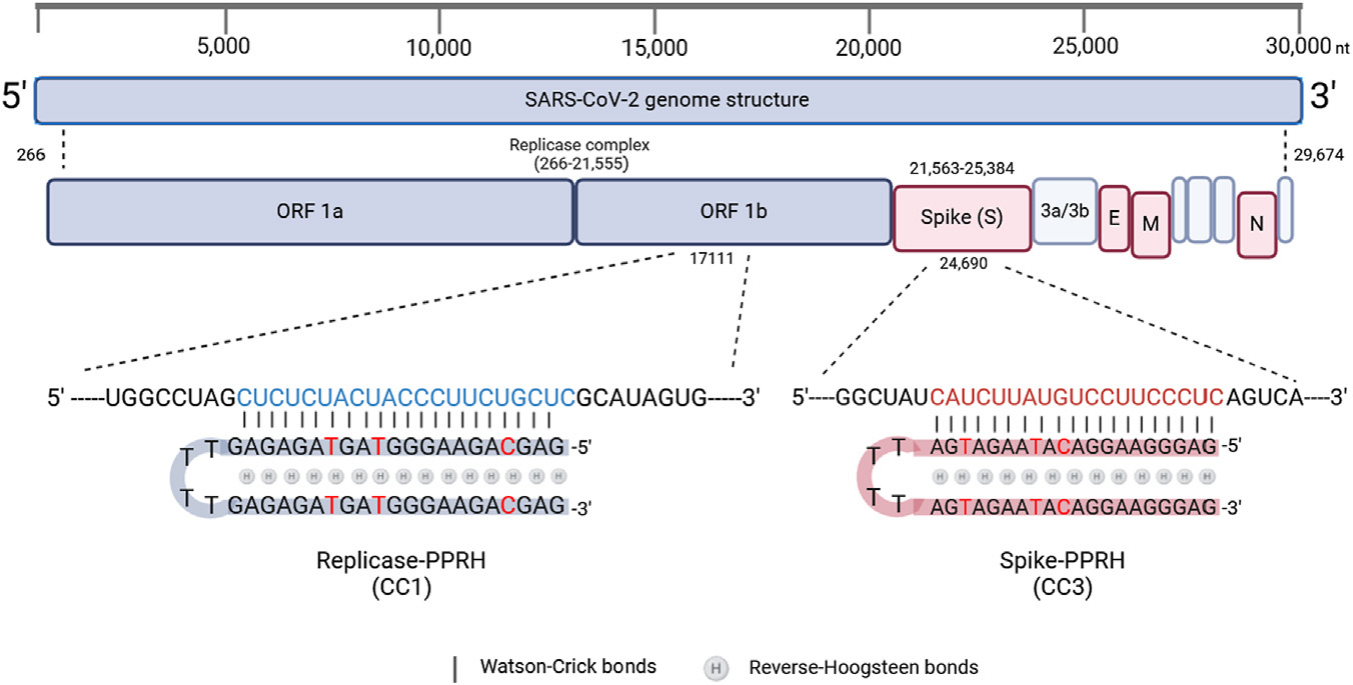
**Schematic representation of the SARS-CoV-2 genome and the location of the target regions for CC1 and CC3 PPRHs.** In blue lettering, the target of CC1-PPRH is in the replicase complex, ORF1b; in red lettering, the target of CC3-PPRH is in the *spike* gene.

CC1-PPRH (CC1) and CC3-PPRH (CC3) target replicase (CTCTCTACTACCCTTCTGCTC), and spike (TCATCTTATGTCCTTCCCTC) regions in the SARS-CoV-2 genome, located at 17,111 and 24,690 positions, respectively (25) (Fig. 1). These sequences were designed by combining the Triplex-Forming Oligonucleotide (TFO) search tool and the following criteria: no more than three pyrimidine interruptions (23), a minimum of 40% G content, and a minimum length of 20 nucleotides. As a negative control, we designed a scrambled PPRH with similar length, G content, and number of interruptions as CC1 and CC3 PPRHs (Table 1). The formation of the hairpin structure for these sequences was previously confirmed (26).

**Table 1:** Oligonucleotide sequences used in this work

Name, location, length, G content and sequence of the oligonucleotides used in this study. These include the PPRHs targeting specific SARS-CoV-2 genome regions (CC1 and CC3), the ASOs and Parallel-Orientation-ASOs targeting the same sequences as CC1 and CC3 PPRHs, oligonucleotides used for RT-qPCR quantitation, VP7 for internalization, and the scrambled PPRH (SCR) used as a negative control.

### PPRHs binding to SARS-CoV-2 target sequences

**Figure 2:**
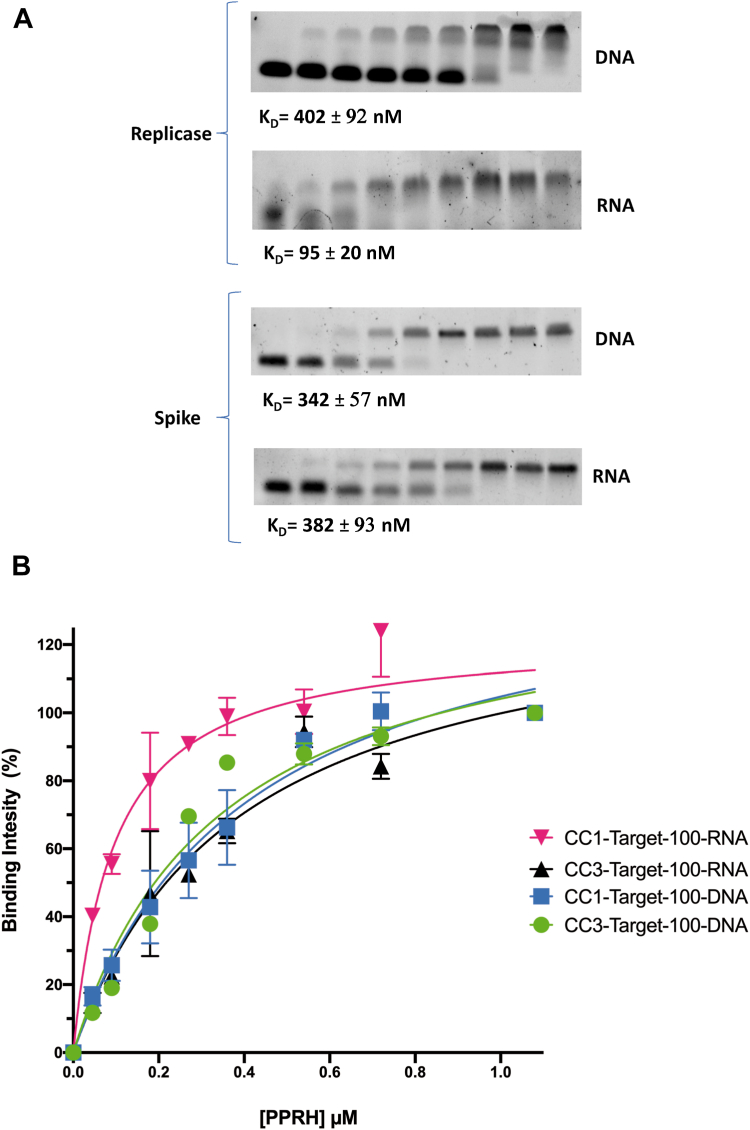
**CC1 and CC3 PPRH binding to their corresponding SARS-CoV-2 genome target sequences.***A*, representative images of the binding assays with CC1 and CC3 PPRHs targeting replicase and spike regions of SARS-CoV-2 and their corresponding constants of dissociation (*Kd*). *B*, binding curves for RNA-PPRH and DNA-PPRH triplex formation are represented as the mean ± SEM of three independent experiments. *Kd*s values were calculated after fitting the binding data as nonlinear regression saturation binding (One site-specific binding) using the GraphPad Software, Prism v. 9.0.1.

To study the interactions between the designed PPRHs and their target regions in SARS-CoV-2, we conducted electrophoretic mobility shift assays (EMSAs) on native gels (Fig. 2). A fixed amount of each target, either as single-stranded (ss) DNA or RNA sequences, was incubated with increasing amounts of the specific PPRH hairpins. In all cases, we observed a shifted band corresponding to the formation of a triplex structure, which increased in intensity in parallel with the amount of PPRH (Fig. 2*A*). The evidence of triplex formation by both PPRHs when binding to their targets was performed in previous work by circular dichroism (26). Binding curves were drawn using the values of the quantification for the triplex bands and the concentrations of PPRH (Fig. 2*B*). The model to fit the binding data was a nonlinear regression saturation binding (One site-specific binding) using the GraphPad Software, Prism v. 9.0.1. The calculated values for the dissociation constants (*Kd*) are indicated in nanomolar in Figure 2*A*.

### CC1-PPRH internalization in VERO-E6 cells

**Figure 3:**
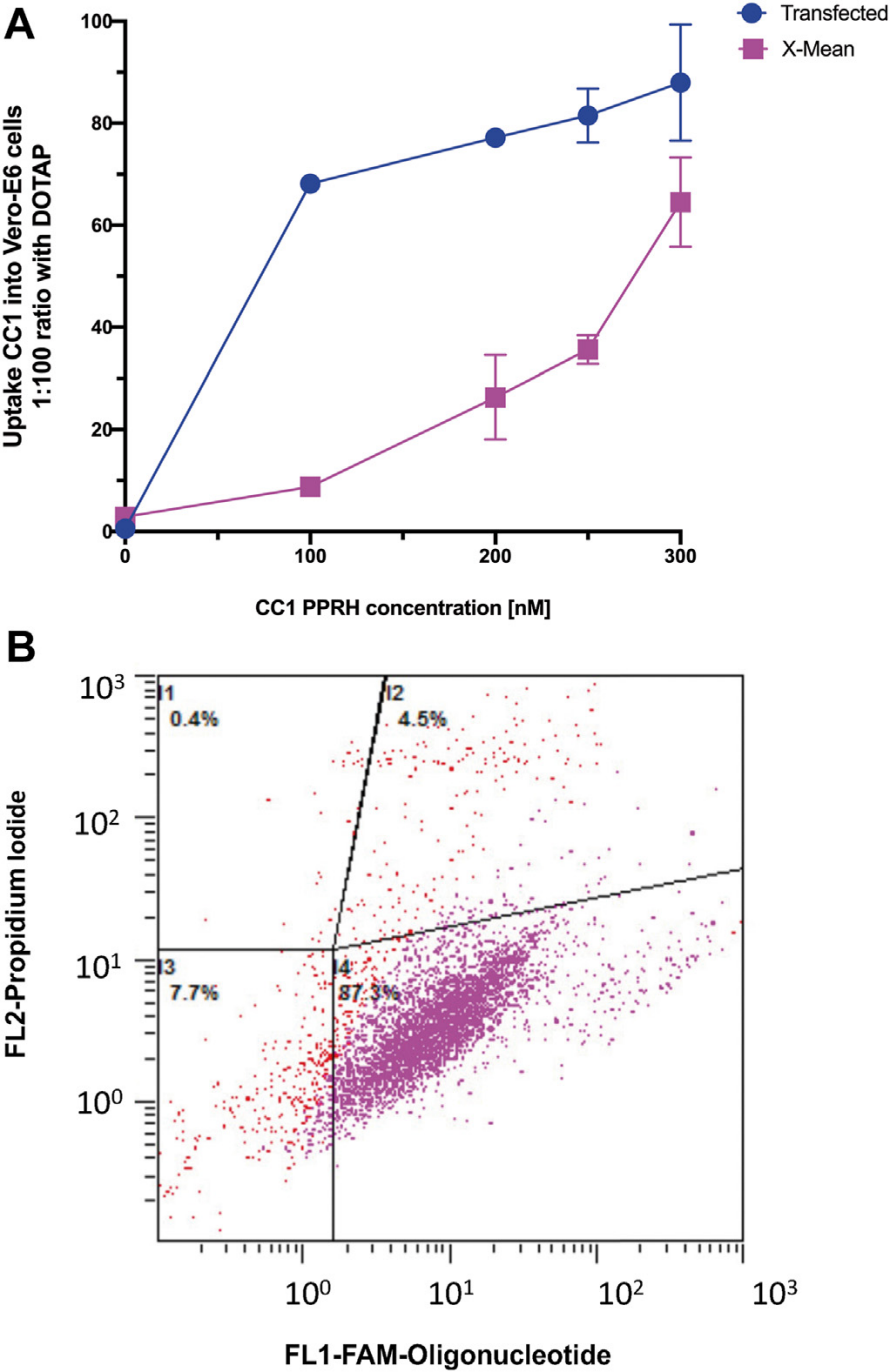
**Cellular uptake of CC1-PPRH in VERO-E6 cells.***A*, uptake of FAM labeled CC1-PPRH in VERO-E6 cells (60,000) determined by flow cytometry. The percentage of transfected cells is shown in blue and the fluorescence X-mean in fuchsia for each concentration of PPRH tested, is represented as the mean ± SD of three independent experiments. *B*, fluorescent dot-plot of CC1 (300 nM) transfected with DOTAP (30 μM) in VERO-E6 cells. I1 non-fluorescent dead cells, I2 fluorescent dead cells, I3 non-fluorescent living cells, and I4 fluorescent living cells.

To evaluate the uptake of PPRHs in cells that express the ACE2 receptor, we used the VERO-E6 cell line (27). Cells were incubated for 24 h with FAM-labeled CC1-PPRH complexed with DOTAP, and internalization was evaluated by flow cytometry. As shown in Figure 3*A*, 70% of the cells were transfected at the minimum concentration of PPRH tested (100 nM) with a fluorescence X-mean value of 10. Optimal internalization occurred in cells transfected with 300 nM of PPRH. At this concentration, 95% of cells showed PPRH internalization with an X-mean value of 70, *i.e.* the amount of PPRH incorporated into the cells (Fig. 3*B*).

### SARS-CoV-2 mRNA proliferation inhibition by CC1 and CC3 PPRHs

**Figure 4:**
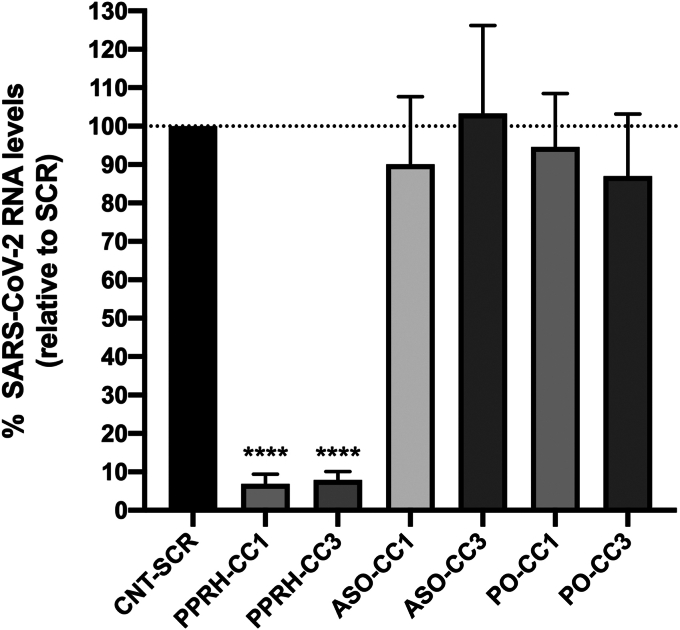
**Levels of viral RNA in VERO-E6 cells infected with SARS-CoV-2.** Cells were transfected with either Scrambled PPRH, specific PPRHs, ASOs, or ASO-POs at a concentration of 300 nM and 30 μM of DOTAP, 24 h before SARS-CoV-2 infection. Levels of SARS-CoV-2 RNA were determined 48 h upon infection by Real-Time PCR. Data represent the mean ± SD of 10 replicates. Statistical significance was analyzed by unpaired *t* test comparing each condition with the CNT-SCR; ∗∗∗∗*p* < 0.0001.

To study the protective effect of the PPRHs upon viral infection, VERO-E6 cells were transfected with different oligonucleotides 24 h before viral infection. SARS-CoV-2 RNA levels were determined 48 h after infection. CC1 and CC3 targeting-PPRHs, antisense oligonucleotides (ASO), parallel orientation ASOs (PO) which have the same orientation as the target, and a scrambled PPRH (SCR-CNT) as a negative PPRH control, were used at a concentration of 300 nM, complexed with 30 μM of DOTAP (Fig. 4). CC1 and CC3 PPRHs reduced SARS-CoV-2 replication by 95 and 94%, respectively (Fig. 4). Successful inhibition of SARS-CoV-2 infection *in vitro* using ASOs had been previously reported (14, 15). However, in our conditions, when using ASOs targeting the same regions as the PPRHs, they showed similar non-inhibitory results as the scrambled PPRH. Altogether, these results revealed that PPRHs were much more efficient than ASOs to target SARS-CoV-2 replication.

### Internalization of CC1 and CC3 PPRHs in K18-hACE2 mouse lung cells

**Figure 5:**
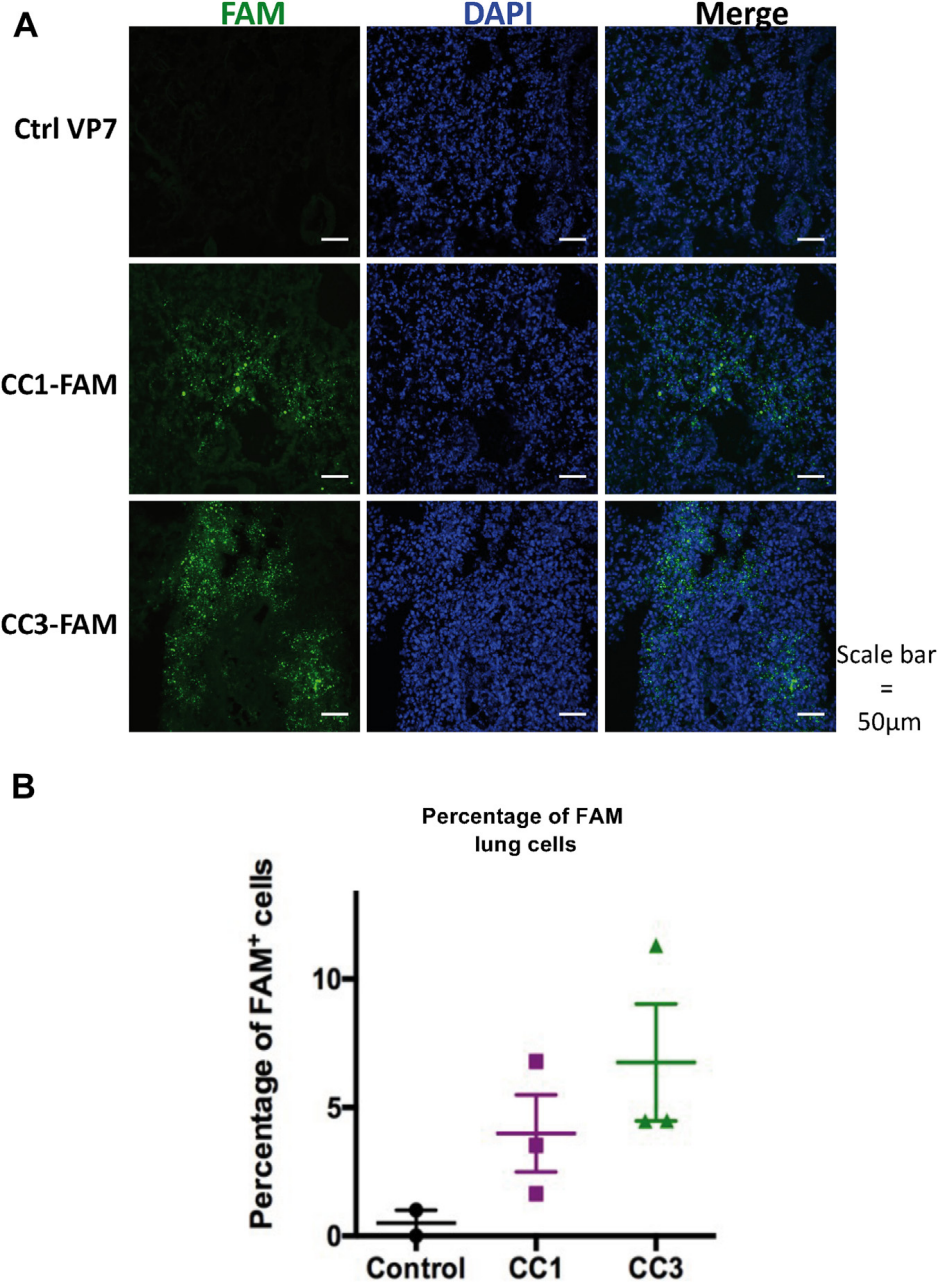
**PPRH internalization in mouse lung cells.** VP7, a control non-fluorescent oligonucleotide and FAM-labeled, CC1 and CC3 PPRHs, were administered intranasally to K18-hACE2 mice. Lung cell fluorescence was evaluated 6 hours after oligonucleotide administration. *A*, images obtained by confocal microscopy. FAM-positive cells are shown in *green*, cells nuclei counterstained with DAPI are shown in *blueB*, quantification of FAM-positive transfected positive lung cells by flow cytometry upon CC1 or CC3 intranasal administration. Experiments were performed in triplicate.

To explore the effects of CC1 and CC3 *in vivo*, K18-hACE2 transgenic mice which express the human ACE2 receptor under the control of the human keratin 18 promoter in the epithelia were used (28, 29). Firstly, we explored the internalization of SARS-CoV-2 targeting PPRHs in mouse lung cells. VP7, a control non-fluorescent oligonucleotide, and FAM-labeled PPRHs CC1 and CC3, were applied intranasally mixed with *in vivo*-JET-PEI. Six hours upon administration, the right lung was snap-frozen in OCT for histology studies, and the left lung was homogenized to obtain a single-cell suspension that was analyzed by flow cytometry for green fluorescence. The 6-h time point was chosen since *in vitro* experiments it is enough to allow for DNA uptake (30). As shown in Figure 5, mice lung cells were successfully transfected with both CC1 and CC3 PPRHs. It can be observed that the fluorescence corresponding to CC1 and CC3 was rather punctuated, suggesting that they were located primarily in the endosomes as it has already been reported for cationic PEI-polyplexes (31, 32). The mean of CC1 and CC3 FAM-positive transfected lung cells was 4.5% and 7%, respectively, as assessed by flow cytometry. On the other hand, control cells, treated with VP7, displayed minimal or no fluorescence (Fig. 5).

### SARS-CoV-2 proliferation inhibition by CC1 and CC3 PPRHs in K18-hACE2 mice

**Figure 6:**
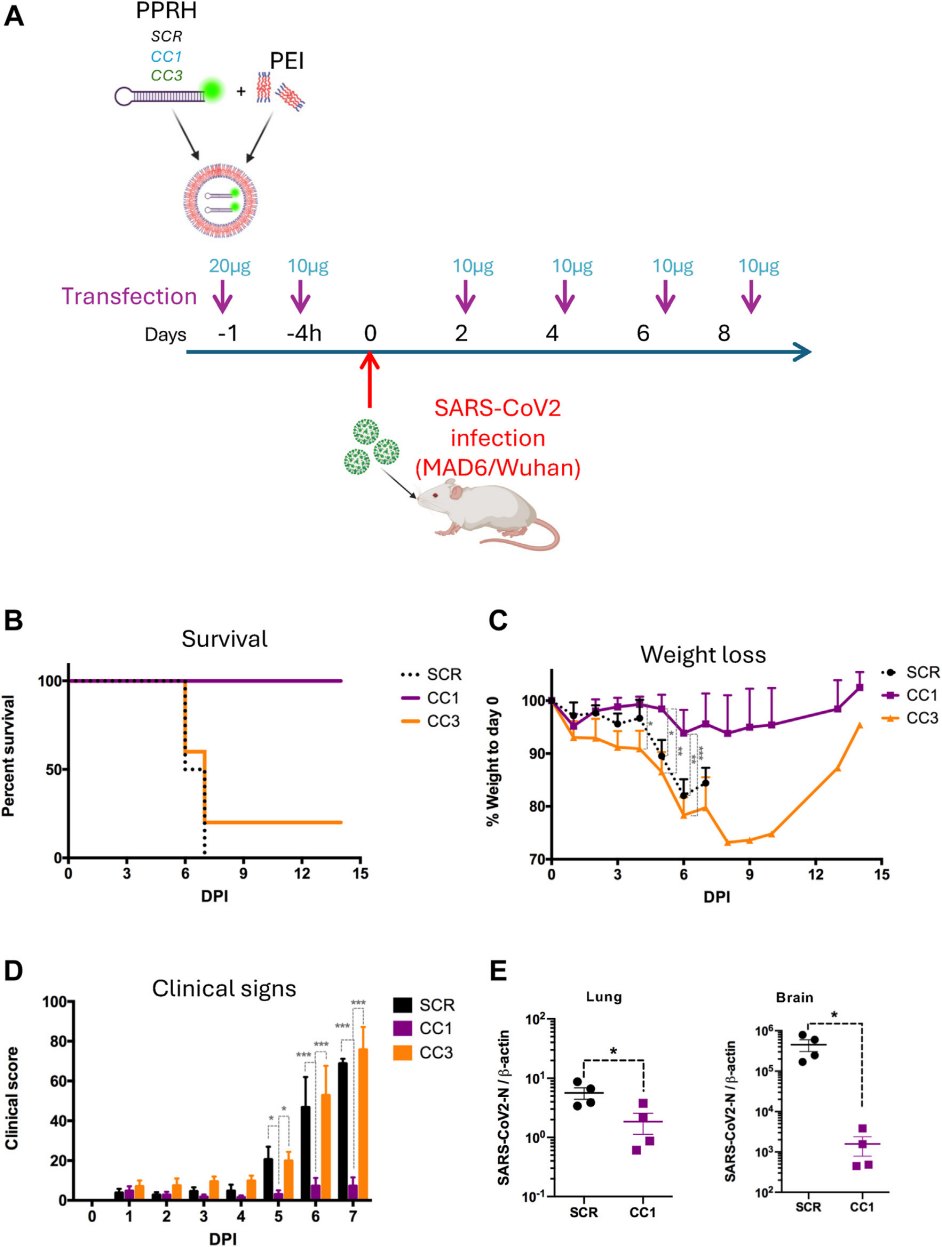
**Effect of CC1, CC3 and SCR PPRH intranasal administration in K18-hACE2 mice infected with SARS-CoV-2.***A*, experimental design of PPRH intranasal administration. The *red* arrow indicates the intranasal inoculation of SARS-CoV-2 (1 x 10^5^ PFU/mouse). *B*, rate of survival of K18-hACE2 mice infected with SARS-CoV-2 and treated with PPRHs, either SCR in *black*, CC1 in *purple* or CC3 in *orange* (C) Mice body weight as assessed daily. Data correspond to weight loss normalized to day 0. *D*, clinical signs in infected mice. Mice were monitored daily and scored. Statistical significance was analyzed by two-way ANOVA with FISHER’s LSD post-test. ∗*p* < 0.05; ∗∗*p* < 0.01; ∗∗∗*p* < 0.001. *E*, viral loads were assessed in the lung and brain of infected animals at day 7 post-infection. RT-qPCR for SARS-CoV2-N were performed as previously described ((33). SARS-CoV2-N expression was normalized to β-actin expression in tissue. ∗*p* < 0.05; unpaired Student’s *t* test. (Panel A created with BioRender).

To evaluate the therapeutic effects of PPRHs against SARS-CoV-2 infection *in vivo*, CC1, CC3, and SCR PPRHs were delivered intranasally using *in vivo*-JET-PEI to K18-hACE2 mice. The administration was performed twice at 20 μg and 10 μg, respectively, 24 and 4 h prior to SARS-CoV-2 infection. Then, mice were challenged with the SARS-Cov2 MAD6 strain, and additional doses of 10 μg of PPRH were administered on days 2, 4, 6, and 8 after infection (Fig. 6*A*). Mice were weighed daily and monitored for 14 days. When the animal presented signs of severe suffering with clinical scores higher than 50 [34], euthanasia was performed. Mice treated with the scrambled control PPRH suffered severe body weight loss (15%), and average clinical signs score of 70 7 days post-infection (dpi) (Fig. 6, *B*–*D*). In the case of CC3, 20% of mice survived the infection (Fig. 6*B*). The other 80% presented weight loss (25%) and evident clinical signs (clinical score 79) over the period of monitorization and were sacrificed 6 to 7 dpi (Fig. 6, *B*–*D*). 14 days after the infection, the survivor mice regained their lost weight (Fig. 6*B*). In contrast, all mice treated with CC1 PPRH survived the infection (Fig. 6*B*), showing no significant body weight lost (less than 5%) (Fig. 6*C*), and only mild or no clinical signs over the 14-day period of monitorization (Fig. 6*D*). To confirm CC1 efficacy, we repeated the PPRH transfection and SARS-CoV2 infection experiment with CC1 and SCR to evaluate the viral load in the lung and brain, two target organs of the infection in K18-hACE2 mice ((33). In this study, viral loads were assessed in lung and brain homogenates of infected animals at day 7 post-infection. CC1 treatment significantly decreased viral burden in the lungs and brain of infected animals, which indicated that CC1 not only restricted viral replication in the primary infection site (lungs), but it also limited viral spread to the brain.

## Discussion

In the current project, we utilized PPRHs as a therapeutic and protective tool against SARS-CoV-2 in *in vitro* and *in vivo* models expressing the ACE2 receptor, to prevent viral proliferation upon infection, disease symptoms and spread. We used two PPRHs, CC1 and CC3, targeting replicase and spike SARS-CoV-2 regions, previously designed for diagnosis of SARS-CoV-2 in human samples (25). PPRH hairpins have already demonstrated their therapeutic properties as a gene silencing tool both *in vitro* and *in vivo* (34, 35, 36, 37). Previously, therapeutic oligonucleotides such as siRNAs (38, 39, 40, 41), ASOs (14, 15) and CRISPR-based systems (17, 18) have shown their ability to suppress SARS-CoV-2 replication. However, some therapeutic strategies explored against SARS-CoV-2 involve invasive systemic delivery by intravenous administration (39). Other strategies, especially for respiratory diseases, are inhaled treatments which can ensure a high drug concentration in lung and blood at low doses (42). In 2022, Zhu, C. *et al.* (15) demonstrated that daily intranasal administration of ASOs targeting SARS-CoV-2 in K18-hACE2 mice presented high antiviral efficacy with no immunogenicity. Similar studies in 2023 showed the inhibition of SARS-CoV-2 replication by administrating siRNAs intranasally (41).

Strategies using chemically modified and stabilized siRNAs against SARS-CoV-2 in VERO-E6 cells showed inhibition of up to 70% of different SARS-CoV-2 at a concentration of 30 nM (41). A similar strategy was implemented by transfecting locked nucleic acid (LNA) ASOs that targeted SARS-CoV-2 Nucleocapsid and Spike regions in Huh-7 cells, which showed a reduction of more than 99% of SARS-CoV-2 replication at 100 nM (15). In the present work, PPRHs demonstrated to have a great effect at 300 nM, preventing viral replication *in vitro* by more than 92%. On the other hand, the ASO versions of CC1 and CC3 PPRHs did not prevent viral replication. These results agree with previous studies that demonstrated that PPRHs have inhibitory effects at concentrations 10 times lower than those needed for ASOs, and at similar concentrations as siRNAs (20). Additionally, the designed PPRHs bound specifically to their intended targets with a low *Kd*. This high affinity led to inhibition of SARS-CoV-2 proliferation both *in vitro* and *in vivo*.

We also explored the *in vivo* effects of PPRHs by the non-invasive intranasal administration route. Out of the two PPRHs tested, CC1-PPRH strongly protected mice from viral spread and disease development when administered prophylactically at 20 and 10 μg, assuring the survival of mice with low clinical signs and weight loss. CC1 and CC3 PPRHs were designed to target the original Wuhan strain of SARS-Cov2, and even if the viral strains used in the experiments *in vitro* and *in vivo* presented here are different, the reported mutations lay outside the target sequences for both PPRHs https://www.ecdc.europa.eu/en/covid-19/variants-concern (Accessed September 16, 2024). Thus, the fact that CC1-PPRH was more effective *in vivo* than CC3-PPRH could be due to other factors. Although the *Kd* of CC1-PPRH is 4 times lower than that of CC3-PPRH, the difference in effectiveness could be more likely due to the viral genes they target. CC3-PPRH targets the spike gene, limiting the expression of the protein and preventing the adequate formation of nascent viral particles. This strategy is effective *in vitro* probably due to the high transfection efficacy in cell lines; however, our data indicate that it is clearly less effective *in vivo*, in a situation in which transfection efficacy is lower. CC1-PPRH targets the Replicase region of the virus, which would stop viral replication and consequently shut down the production of viral particles. As shown by our data, this strategy is more effective *in vivo* as it probably slows down viral replication and gives time to the infected host to mount an adequate immune response to the infection. siRNA targeting the replicase region has been shown to limit *in vitro* and *in vivo* viral replication (39) thus confirming that targeting the replication machinery is suited for antiviral development against SARS-CoV2. Other studies administrating intranasally LNA ASOs demonstrated that they can be effective in either prophylactic or post-infection treatments. Zhu and collaborators (15) found that mice treated with daily intranasal administrations of 400 μg of naked LNA ASOs presented no weight loss until 4 days post-infection (dpi). However, after 4 days, weight loss was significant and only a small group of mice survived viral infection (34). Daily intranasal administration of modified siRNAs (40 μg) showed low but significant decrease of viral proliferation in lungs at 7 dpi (41). Intravenous administration of 20 μg of siRNA with lipid nanoparticles led to 20% mice survival 7 dpi (39). Both ASOs (15) and siRNAs (39) strategies showed low or no significant immune stimulatory effects. Previous *in vitro* studies comparing the immunogenicity induced by PPRHs and siRNAs demonstrated that PPRHs did not generate an immune response, while the transfection of siRNAs induced unintended immune reactions (24). Additionally, studies in mice showed that intranasal administration of *in vivo*-JET-PEI alone showed no significant immunogenic response (43). Given the low impact of CC1-PPRH on mice weight and its protective effects in terms of clinical signs, we could conclude that intranasal administration of PPRHs complexed with *in vivo*-JET-PEI in mice is unlikely to produce an unwanted immune response.

When the COVID-19 pandemic started over 4 years ago, the development and deployment of therapies emerged as main strategies to mitigate the impact of the virus (44). Here we explored the PPRH technology as a protective barrier against SARS-CoV-2 infection. Although vaccines are usually the priority agents to prevent the spreading of infectious diseases, their development is time-consuming and undergoes many steps before being approved and commercialized. Out of 273 vaccine candidates, 108 had entered the human clinical phase and just four reached phase 4 (45). In the case of SARS-CoV-2, vaccines did not fully block the infection but helped control the clinical presentation of the disease, preventing the characteristic cytokine storm that often leads to the most severe adverse effects produced by the infection (46). Antiviral treatments based on oligonucleotides could therefore offer complimentary targeted therapeutic tools to control the disease in unvaccinated patients, or in patients with receding immunity to the virus. Since other oligonucleotide-based strategies have showed an effective inhibitory effect against SARS-CoV-2, we consider PPRHs as a potential therapeutic tool for viral infection. Our *in vitro* and *in vivo* findings suggest CC1-PPRH as a potential candidate against SARS-CoV-2. This PPRH demonstrates the ability to protect from infection and viral spread. PPRHs present many advantages over other therapeutic oligonucleotide competitors, such as their efficacy and their inexpensive synthesis given their non-modified DNA nature (24), indicating their possible production at large scale in a new viral pandemic context. Although further studies are needed before implementing PPRH in clinical assays, our technology could potentially be used to protect patients at risk of developing SARS-CoV-2 infection and as a treatment for the infection. Our findings indicate that PPRHs offer promising approaches to improve the use of oligonucleotides in biomedical applications, particularly in the field of viral diseases.

## Conclusion

The main conclusions are that CC1-PPRH and CC3-PPRH directed against the Replicase and Spike RNA regions of the SARS-CoV-2 virus, respectively, can inhibit viral proliferation when transfected into VeroE6 cells infected with SARS-CoV-2. Importantly, when administrated intranasally into K18-hACE2 mice, CC1-PPRH inhibits SARS-CoV-2 replication in target organs, protects transgenic mice from the disease and decreases the clinical signs of the infection.

## Experimental procedures

### Oligonucleotides

We used previously two designed PPRHs against SARS-CoV-2 replicase and spike regions, named CC1 and CC3, respectively (25), with arm lengths of 20 or 21 nt among the eight possibilities identified to target the viral genome with a minimal length of 17 nt (26). According to the predicted secondary structure for SARS-CoV-2 RNA described by Lan *et al.* (47) CC1 target is a polypyrimidine sequence near a large potential hairpin with a 3 nt-bulge (6 unpaired nt out of 21 nt) whereas CC3 target is a polypyrimidine sequence involved in a potential hairpin loop that cover nine unpaired nt of a 11 nt loop and two bulges (11 unpaired nt out of 20 nt). Additionally, we designed a scrambled PPRH (SCR) as a negative control. The designed PPRHs were synthesized as non-modified oligodeoxynucleotides by Sigma-Aldrich, resuspended in sterile Tris-EDTA buffer (10 mM Tris and 1 mM EDTA, pH 8.0) from Sigma-Aldrich, and stored at −20 °C. For SCR PPRH, we performed BLAST analyses to avoid unintended mismatches. We also designed and tested ASOs directed against the same sequences as CC1 (5′-GAGCAGAAGGGTAGTAGAGAG-3′) and CC3 (5′-GAGGGAAGGACATAAGATGA-3′), and their parallel orientation (PO) counterparts were used as negative controls PO-CC1 (5′-GAGAGATGATGGGAAGACGAG-3′) and PO-CC3 (5′-AGTAGAATACAGGAAGGGAG-3′). Non-fluorescent VP7 oligonucleotide (5′-CGCGATCCATGGACACTATCGCTGCAAG-3′) was used as a negative control to determine FAM-PPRH incorporation into mice lung cells.

### Cell culture

Vero E6 cells (mycoplasma-free), derived from the African green monkey kidney, were obtained from the Cell Bank of the Institute of Neurosciences, Autonomous University of Barcelona and grown as described in (45), in Dulbecco’s Modified Eagle Medium (DMEM) (Sigma Aldrich, Boston) supplemented with 5% fetal bovine serum (FBS) (Logan, UT, USA) or in Ham’s F12 medium supplemented with 10% FBS (GIBCO, Invitrogen).

### PPRHs transfection

Cells were plated in 6-well dishes in 900 μl of Ham’s F12 medium supplemented with 10% FBS. For transfection we used a mixture of 1,2-Dioleoyl-3-trimethylammonium propane (DOTAP; Biontex, Germany) with variable quantities of PPRHs always maintaining a molar 1:100 ratio of PPRH:DOTAP in serum-free medium up to 100 μl. After a 20-min incubation at room temperature, the mixture was added to the cells to reach a final volume of 1 ml.

### Fluorescent microscopy and flow cytometry

Cells (100,000) were plated in Ham’s F12 medium in 6-well dishes and transfected the following day with 10 to 30 μM of DOTAP and 100 to 300 nM of CC1 PPRH labeled with fluorescein (6-FAM) in its 5′-end. Twenty-4 hours following transfection, cells were harvested through trypsinization, resuspended in PBS, and then centrifugated at 800*g* at 4 °C for 5 min. The resulting pellet was resuspended in 400 μl of cold PBS. Prior to flow cytometry analyses, propidium iodide (Merck) was added to a final concentration of 5 μg/ml. Flow cytometry analyses were conducted in a Gallios flow cytometer (Beckman Coulter, Inc) to detect green and orange fluorescences of both control and treated cells.

### Electrophoretic mobility shift assay (EMSA)

Electrophoretic mobility shift assays (EMSA) were performed with 6-FAM-labeled single-stranded DNA (ssDNA) or RNA probes corresponding to the SARS-CoV-2 targets and their corresponding PPRHs, in a buffer containing 10 mM MgCl2, 100 mM NaCl, and 50 mM HEPES (pH 7.2), supplemented with 5% glycerol. Binding reactions were performed with increasing amounts of CC1 and CC3 PPRHs, from 0 to 300 ng combined with a fixed amount, 100 ng, of 6-FAM labeled probes. As a negative control, 100 ng of a scrambled PPRH (SCR: AGAGAGGTTAGGAGGACAAGGTTTTGGAACAGGAGGATTGGAGAGA) was used. The binding reactions (20 μl) were incubated for 30 min at 37 °C. Electrophoreses were carried out on non-denaturing 8% polyacrylamide gels containing 10 mM MgCl_2_, 5% glycerol, and 50 mM HEPES (pH 7.2), at a constant voltage of 190 V at 4 °C, using a running buffer of 10 mM MgCl_2_ and 50 mM HEPES (pH 7.2). Bands were visualized using the Gel DocEZ with Image Lab Software, Version 6.0 (Bio-Rad). All reagents were purchased from Sigma-Aldrich. The intensity of the bands was quantified using the ImageJ2 software, Version 2.9.0.

### Virus infection and quantification

After 24 h of transfection with PPRH:DOTAP complexes, Vero E6 cells were infected with 200 plaque-forming units (PFU) of the SARS-CoV-2 strain hCoV19/Spain/SP-VHIR.02, D614G(S). After 48 h, RNA was extracted from the supernatants using the Quick-RNA Viral Kit from Zymo Research. Quantification of SARS-CoV-2 production was performed by qPCR using the qScript XLT One-Step RT-qPCR ToughMix with ROX (Quanta Biosciences). This included the specific probe 2019-nCoV_N1-P (5′-FAM-ACCCCGCATTACGTTTGGTGGACC-BHQ1-3′), as well as primers 2019-nCoV_N1-F (5′-GACCCCAAAATCAGCGAAAT-3′) and 2019-nCoV_N1-R (5′-TCTGGTTACTGCCAGTTGAATCTG-3′) obtained from Biomers (Ulm, Germany).

### Animals

B6Cg-Tg(K18-hACE2)2Prlmn/J mice (Charles River Laboratories, France) were employed in protection experiments. All aspects of this study were approved by the office of Environmental Health and Safety at CISA-INIA-CSIC, Madrid, Spain before the initiation of this study. Ethical requirements were approved by the Consejo Superior de Investigaciones Científicas (CSIC) Ethics Committee and the Comunidad Autónoma de Madrid (PROEX 295.6/21). The animals were generally housed in groups of five, always following the space requirements specified in legislation (EU Directive 2010/63 and Spain regulation RD53/2013, modified by RD1386/2018). Experimentation with infected mice was carried out in BSL3+ laboratories (CISA-INIA-CSIC). All animals received food and water ad libitum. Animal welfare measures were applied, considering replacement, reduction, and refinement. Environmental enrichment was implemented. Animals were anesthetized with isofluorane (3% for induction, 1.5% for maintenance) before the intranasal administration of PPRHs. To study PPRH delivery to lung cells, intranasal transfection of 20 μg FAM-labeled PPRHs with *in vivo*-jetPEI (Polyplus, France) was performed at an N/P ratio of 12. As fluorescence control, transfection was performed with a non-fluorescent oligonucleotide derived from bluetongue virus VP7 sequence. Animals were sacrificed 6 h post-administration, lungs extracted, and one lobe cryopreserved in OCT to determine tissue transfection in tissue cryosection (10 μm) counterstained with 4′,6-Diamidine-2′-phenylindole dihydrochloride (DAPI) (Sigma). Images were captured using confocal microscopy (Zeiss Airyscan 880) and processed with ImageJ software (http://rsbweb.nih.gov/ij/US National Institutes of Health). The second lobe was mechanically disaggregated and digested with collagenase (0.5 mg/ml) for 1 h at 37 °C. A single-cell suspension was then obtained after filtration through a 70 μm cell strainer. Dead cells were excluded from the analysis using the viability marker 7-Aminoactinomycin D (7-AAD) (BD Pharmingen). Samples were acquired using a FACSCelestaSORP flow cytometer (BD Biosciences), and data were analyzed with FlowJo software (BD Biosciences). The percentage of FAM–positive cells was determined on live cells after doublet exclusion.

### SARS-CoV-2 infectious challenge and viral load assessment in target organ homogenates

To perform the *in vivo* challenge experiments, the SARS-CoV2 MAD6 strain (kindly provided by Dr Luis Enjuanes, CNB, Madrid, Spain), was used since it has been established to cause the disease in K18-hACE2 mice (33) and belongs to the same Wuhan-Hu-1 lineage as the strain used in the *in vitro* experiments hCoV19/Spain/SP-VHIR.02, D614G(S). The sequences targeted by PPRHs CC1 and CC3 are identical for both viral isolates. K18-hACE2 mice were challenged with 10^5^ PFU of MAD6 SARS-CoV-2 by the intranasal route after two doses of intranasal PPRH transfection, 24 and 4 h before the viral infection (20 μg and 10 μg, respectively, complexed with *in vivo*-jetPEI at a N/P ratio of 12). Transfection with PPRH was then repeated with 10 μg on Days 2, 4, 6, and 8 post-infection. Body weight and clinical scores were followed daily in five K18-hACE2 mice per group (SCR control, CC1 and CC3) for each experiment. Mice were observed and weighed daily post-challenge, and clinical signs were scored according to (33). The sum score in clinical signs (based on body weight, appearance, motility, and respiration) was used to evaluate disease severity. Euthanasia was applied when signs of severe disease burden and suffering (clinical score higher than 50) were detected. Target organs (lungs and brain) were collected at day 7pi from SCR- or CC1-transfected mice and homogenized using a 2 min homogenization cycle in a tissueLyser II (Qiagen) at maximum frequency (30 Hz). Homogenate RNA was obtained using the IndiSpin Pathogen extraction kit (Indical) following the manufacturer’s instructions and stored at −80 °C until use. Viral load in tissue homogenates was assessed by RT-qPCR as described in (33). For representation, SARS-CoV2-N1 transcript levels were normalized to β-actin transcript content.

### Statistical analyses

Statistical analyses were carried out using GraphPad Prism 6 (GraphPad Software, CA, USA). Data represented the mean value and the standard deviation of the mean (SD) from at least three separate experiments. Levels of statistical significance are indicated as follows: *p* < 0.05, *p* < 0.01, *p* < 0.001, or *p* < 0.0001 (∗∗∗∗).

## Data availability

Dataset available on request from the authors.

## Conflicts of interest

The authors declare that they have no conflicts of interest with the contents of this article.
